# The hematin-dihydroartemisinin adduct mobilizes a potent mechanism to suppress **β**-hematin crystallization

**DOI:** 10.1016/j.jbc.2025.110310

**Published:** 2025-05-29

**Authors:** Hamidreza Azargoshasb, Huan-Jui Lee, David J. Sullivan, Jeffrey D. Rimer, Peter G. Vekilov

**Affiliations:** 1 William A. Brookshire Department of Chemical & Biomolecular Engineering, University of Houston, Houston, Texas, USA; 2 Welch Center for Advanced Bioactive Materials Crystallization, University of Houston, Houston, Texas, USA; 3 Department of Molecular Microbiology and Immunology, Johns Hopkins School of Public Health, Baltimore, Maryland, USA; 4 Department of Chemistry, University of Houston, Houston, Texas, USA

## Abstract

Malaria remains a significant public health challenge in equatorial regions of the world, largely owing to the parasite's emerging resistance to the recently introduced drugs of the artemisinin (ART) family. In the human body, most ART-derivative drugs are metabolized to dihydroartemisinin (DHA), which, in the parasite, after activation by heme, can form a hematin–dihydroartemisinin adduct (H-DHA). Here we test whether and how H-DHA inhibits hematin crystallization, the main constituent of the heme detoxification pathway of malaria parasites. We find that H-DHA is a poor inhibitor of classical crystal growth—it weakly blocks the growth sites on crystal surfaces—and, counterproductively, a promoter of **β**-hematin nucleation, driven by a boost in the formation of precursors. We establish that at elevated hematin concentrations, H-DHA activates two nonclassical pathways that transform it into a potent **β**-hematin growth inhibitor. First, **β**-hematin crystallites, whose nucleation is promoted by H-DHA, incorporate into large **β**-hematin crystals and suppress their growth, likely by straining the crystal lattice. A second consequence of H-DHA is the generation of macrosteps on **β**-hematin crystal surfaces that hinder growth. Importantly, the induced growth suppression is irreversible and persists even in the absence of H-DHA. Our findings suggest that a partial resistance mechanism to artemisinin-class drugs in trophozoite-stage parasites may be due to the reduced concentrations of hematin and H-DHA, which deactivate the dual nonclassical mode of action of the adduct in the delayed-clearance parasite strains.

Malaria is a global disease, with the highest burden falling on populations in Africa and South Asia (1). Approximately 3.2 billion people are at risk of infection, and children are particularly vulnerable to the disease (1). Malaria is caused by five species of the *Plasmodium* parasite: *Plasmodium falciparum*, *P. vivax*, *P. malariae*, *P. knowlesi*, and *P. ovale* (2). Among these, *P. falciparum* is the most prevalent and is responsible for the majority of severe cases (3). During the intraerythrocytic stage of its life cycle, *Plasmodium* parasites catabolize host hemoglobin, releasing Fe(II) heme (4, 5, 6, 7). The heme then rapidly oxidizes to Fe(III) hematin, which is highly toxic to the parasite in its free form (8, 9, 10). The primary detoxification mechanism employed by the parasite is the sequestration of hematin into non-toxic crystalline hemozoin (4, 11, 12, 13). Hematin crystallization has been identified as the target of several antimalarial drugs, which suppress crystallization and increase the concentration of free hematin within the parasite to toxic levels (14).

The rise in malaria-related deaths during the last century, largely attributed to chloroquine resistance (15), has been mitigated by the widespread use of artemisinin (ART) combination therapies as the first line of antimalarial defense (6, 16, 17). In human hosts, artemisinin prodrugs, such as artesunate (ARS) and artemisinin (ART), convert to dihydroartemisinin (DHA, Fig. 1, *A*–*C*). Upon entering the parasites, DHA is activated by freshly released Fe(II) heme. The resulting cleavage of the endoperoxide bridge (Fig. 1*C*) generates free radicals that damage essential proteins and lipids (18, 19), reducing parasite density by a dramatic 10,000-fold (20, 21, 22, 23).

**Figure 1:**
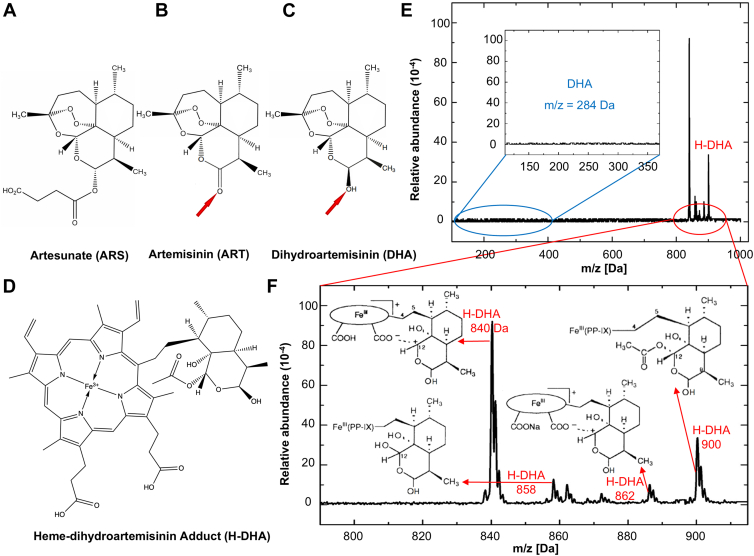
**Identification of the heme-dihydroartemisinin adduct, H-DHA.***A–D*, molecular structures of (*A*), artemisinin, ART; (*B*) artesunate, ARS; (*C*) dihydroartemisinin, DHA; and (*D*) H-DHA. *Red arrows* in (*B*) and (*C*) point at functional groups that distinguish dihydroartemisinin and artemisinin. *E*, the mass spectrum of isolated H-DHA shows characteristic peaks in the range of 840 to 900 Da. Inset: Absence of mass ion peaks associated with the parent drug DHA. *F*, extended view of the 790 to 915 Da m/z range of the mass spectrum in panel (*E*). H-DHA species are formed due to a fragmentation process in mass spectrometry, with m/z values of 840, 858, 862, and 900 Da; the latter corresponds to the molecular mass of H-DHA.

These studies found that the free radicals may also attack the abundant surrounding hematin, yielding a hematin-dihydroartemisinin adduct (H-DHA) (24). Our previous work established that hematin adducts of artemisinin and artesunate, H-ART and H-ARS, are not passive bystanders of hematin crystallization and parasite suppression (25, 26). Time-resolved *in situ* atomic force microscopy (AFM) of growing **β**-hematin (the synthetic analogue of hemozoin (12)) surfaces revealed that H-ART and H-ARS inhibit crystal growth by blocking the kinks on the crystal surface (26). Kinks are critical sites where solute molecules incorporate into the growing crystal (27, 28). This mode of action is similar to how some quinoline-class drugs, such as amodiaquine and mefloquine, operate (29).

The finding of **β**-hematin suppression by adducts of hematin and artemisinins relates to observations with tightly synchronized *P. falciparum* exposed to short drug pulses. These studies revealed a second mode of action of both ART and DHA (30), distinct from their free-radical activity, responsible for an initial high-sensitivity phase during the parasites’ ring stage. A second high-sensitivity phase starts at 24-h parasite age, when the parasites enter their trophozoite stage and hemozoin crystals grow. The second period of high sensitivity suggests that ART and DHA employ a mechanism whereby their hematin adducts suppress hemozoin crystal growth and hematin accumulates above a toxicity threshold (6) owing to this suppression (25, 26); the reasons for the circa 20-fold stronger activity of DHA at this stage have remained elusive.

In the last 15 years, a delayed clearance phenotype (31) has emerged in Southeast Asia and is rapidly spreading in Africa, reversing decades of progress in malaria treatment and undermining malaria control and elimination efforts (32, 33, 34, 35, 36). This resistance has been linked to mutations in the Kelch13 gene (37) and is associated with reduced hemoglobin availability. Initially, the increased tolerance of ring-stage parasites to artemisinin-based treatments (38) was associated with delayed activation and production of free radicals. More recent studies suggest that the delayed clearance may also be related to the hemozoin suppression activity of H-DHA at the trophozoite stage, when hemozoin crystals actively grow (6, 39). The concentration of H-DHA in trophozoites of delayed-clearance *P. falciparum* strains (C580Y and CAM3.11-R539T) is circa 10 μM, lower by about twofold than the values measured in artemisinin-sensitive strains (CAM WT and CAM3.11-Reverant) (6). Measurements of the free hematin concentrations in the four parasite strains establish that at the trophozoite stage, at parasite age between 28 and 35 h, it is also about twofold lower in delayed clearance parasites than in sensitive ones (39).

Here, we address the molecular mechanisms that potentially allow mild reductions of the concentrations of H-DHA and hematin to render delayed-clearance parasites at their trophozoite stage resistant to artemisinin-class drugs. We show that at moderate hematin and H-DHA concentrations, as in the delayed-clearance parasites, H-DHA acts as a weak crystal growth inhibitor, substantially less efficient in suppressing **β**-hematin growth than previously studied antimalarials acting by a similar mechanism, such as mefloquine and amodiaquine (25, 26, 29, 40). At elevated concentrations of hematin and H-DHA, as in the sensitive strains, a powerful mechanism of irreversible inhibition operates that arrests **β**-hematin growth even after the removal of the inhibitor. This mechanism does not operate with H-ARS and H-ART (25) and may be the cause of the strong suppression of trophozoite-stage *P. falciparum* parasites by DHA (30).

## Results and discussion

### Synthesis of the H-DHA adduct

The H-DHA adduct was synthesized by a two-phase reaction between heme(II) and DHA, similar to the method developed to produce H-ART and H-ARS (Figs. S1, S2) (25, 26). We used mass spectrometry to identify the product of the reaction. The mass spectrum of the product shows a major peak at m/z = 900 Da, corresponding to the molecular mass of the H-DHA adduct (Fig. 1, *D* and *E*). The peaks with m/z values of 840, 858, and 862 Da correspond to H-DHA species that are formed by fragmentation during the measurement of the mass spectrum (Fig. 1*F*). These peaks resemble those associated with H-DHA found in extracts from the spleens of mice infected with malaria and treated with ART (24). The difference in the mass peaks between H-ART and H-DHA is 2 Da, which corresponds to the 2 extra hydrogen atoms in H-DHA. Furthermore, we do not see a 284-Da peak from dihydroartemisinin (Fig. 1*E*, inset). The absence of this peak indicates that the purification procedure mostly removes the parent drug and minimizes the possibility of residual drug interfering with inhibition of **β**–hematin crystallization (41).

### Mechanisms of **β**-hematin crystal growth

**β**-hematin is a synthetic crystalline analog to hemozoin. Both natural and synthetic hematin crystals have triclinic lattices with P $\bar{1}$ crystallographic symmetry and exhibit identical morphologies elongated along the [001] axis, with basal faces defined by {100} planes and side {010} faces (42, 43, 44). The digestive vacuole of the *Plasmodium* parasites is filled mostly with a water-based liquid, in which the proteases that process hemoglobin operate (45). It was found that the hemozoin crystals that grow there are suspended in nanospheres comprised of a mixture of mostly five neutral lipids and the nanospheres are crucial for hemozoin crystal growth and hematin sequestration (46). As they are in contact with the main aqueous phase in the digestive vacuole, the mixture of lipids necessarily holds small amounts of dissolved water (27, 47). Further studies addressed the issue of whether water-based crystallization can be an efficient hematin detoxification mechanism and found out that it cannot (27, 48). The main argument is that at the pH of the digestive vacuole, hematin solubility, 2 nM, is so low that, with the rate of hematin release as the parasite feeds on hemoglobin, the solution would be extremely supersaturated, *e.g.*, 10^6^-fold. These studies showed that at these extreme supersaturations, **β**-hematin crystals accumulate numerous defects, and the associated elastic strain fully stalls their growth. With this in mind, following an approach common in pharmacology, we mimic the neutral lipids environment of the parasite’s digestive vacuole and employ as solvent n-octanol saturated with citric buffer at pH 4.8; we have previously referred to this solvent as CBSO (27, 47, 48). Elucidating the molecular mechanisms by which hematin molecules from the solution incorporate into crystals provides a benchmark to highlight the activity of a crystallization inhibitor.

**Figure 2:**
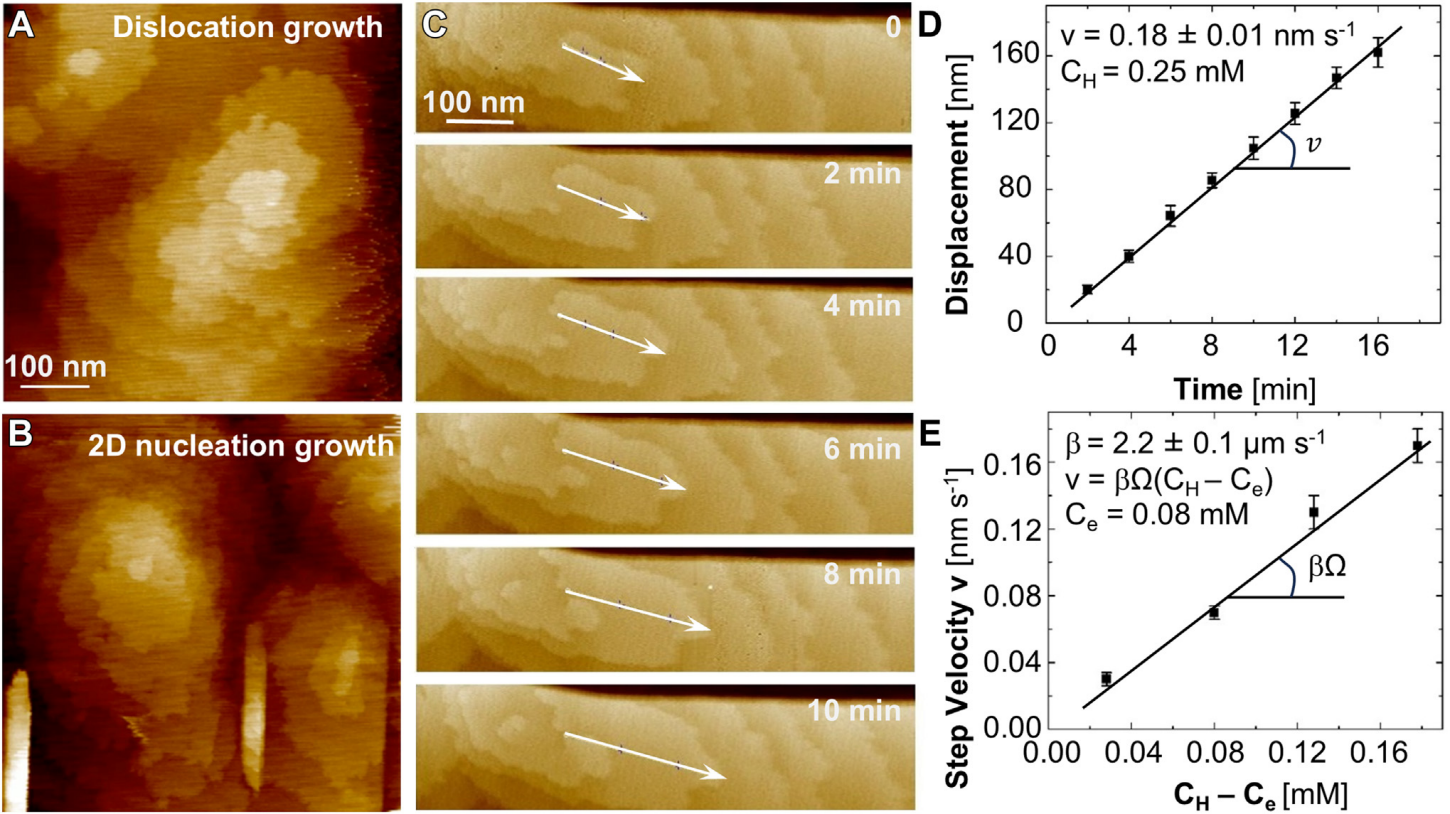
**Hematin crystal growth characterized by time-resolved *in situ* AFM.***A* and *B*, hematin crystal layers, generated by dislocations in (*A*) and 2D nucleation in (*B*). *C* and *D,* evolution along the [001] direction of the location of a dislocation-generated step at C_H_ = 0.25 mM. *Arrows* in (*C*) trace step location that advances owing to growth. Error bars in (*D*) indicate standard deviations from the averaged displacements of five steps in (*C*). The slope is the step velocity v. *E*, step velocity v in the [001] direction as a function of hematin concentration C_H_. Error bars indicate the standard deviations from the mean slopes of the linear correlations as in (*D*). The solubility C_e_ = 0.08 mM. The slope represents the product of the kinetic coefficient β and the molecular volume Ω.

We monitored **β**-hematin crystals of lengths between 10 and 30 μm and widths between 1 and 2 μm by time-resolved *in situ* AFM. We find that the crystals grow classically (49, 50). New layers are generated by both dislocations and 2D nucleation (Fig. 2, *A* and *B*) (25, 26, 27, 28, 29, 40, 51). The heights h of both dislocation- and 2D-nucleation-generated layers are about 1.1 nm, close to the lattice parameter a = 1.22 nm (12). The edges of the unfinished layers, referred to as steps, are the locations where solute molecules incorporate into specially configured sites called kinks (43, 52). Solute incorporation results in the advancement of steps. Thus, the step growth rate, or velocity v, is a direct indicator of the rate of the chemical reaction between kinks and solute molecules. Furthermore, the growth of a layer to cover an entire crystal face thickens the crystal by one lattice parameter, which makes v a prime determinant of the crystal growth rate V normal to the interface and the ability of the crystal to sequester toxic hematin.

The shapes of the dislocation spirals and the 2D islands are similarly elliptical and elongated along the [001] direction. This shape anisotropy reflects the differences in v whereby the steps grow fastest in [001] direction. We measure v by tracking the displacement of individual steps between successive AFM images, with respect to a fixed reference point (Fig. 2*C*). The slope of the correlation between the step displacement and time gives the step velocity v at the hematin concentration $C_{H}$ selected during the measurement (Fig. 2*D*).

We find that v scales linearly with $C_{H}$ and use this correlation to evaluate the solubility $C_{e}$ as the value of $C_{H}$ at which v reaches zero (Fig. S3*A*). The measured solubility $C_{e}$ = 0.08 mM is lower than a previous measurement of 0.16 mM (28). We attribute the lower solubility to a higher purity (97%) source of hematin reagent used here. Purer hematin putatively forms more perfect crystals, which are less strained. The lower elastic energy associated with the diminished strain brings down the chemical potential $\mu_{cr}$ of hematin in the crystals. Since at equilibrium $\mu_{cr}=\mu_{H}=\mu_{H}^{o}+RT\;\ln\;C_{e}$, where $\mu_{H}$ is the chemical potential of hematin in the solution and $\mu_{H}^{o}$ is its standard value, a lower $C_{e}$ directly manifests from lower $\mu_{cr}$ (Fig. S3*B*).

The linear correlation between the step velocity v and the hematin concentration $C_{H}$ is analogous to numerous previous observations with solution-grown crystals (44, 49, 52, 53, 54, 55), including **β**-hematin (27, 28). The steps grow as a result of a bimolecular reaction between solute molecules and kinks (55), and since the kink density is independent of $C_{H}$ (27, 28), *v* is expected to scale linearly with $C_{H}$ (27, 53, 56, 57) According to the relation $v=\beta \Omega \left(C_{H}-C_{e}\right)$, where ***β*** is step kinetic coefficient and *Ω* = 0.708 nm^3^ is the volume occupied by a hematin molecule in the crystal (12). The data on hematin step growth yields ***β*** = 2.2 ± 0.1 μm s^−1^ (Fig. 2*E*). The bimolecular rate constant for binding of solute to a kink (55) $k_{a}=\beta \Omega a^{-1}=$ (1.15 ± 0.05) × 10^3^ M^−1^ s^−1^.

### Hematin crystal growth in the presence of H-DHA

**Figure 3:**
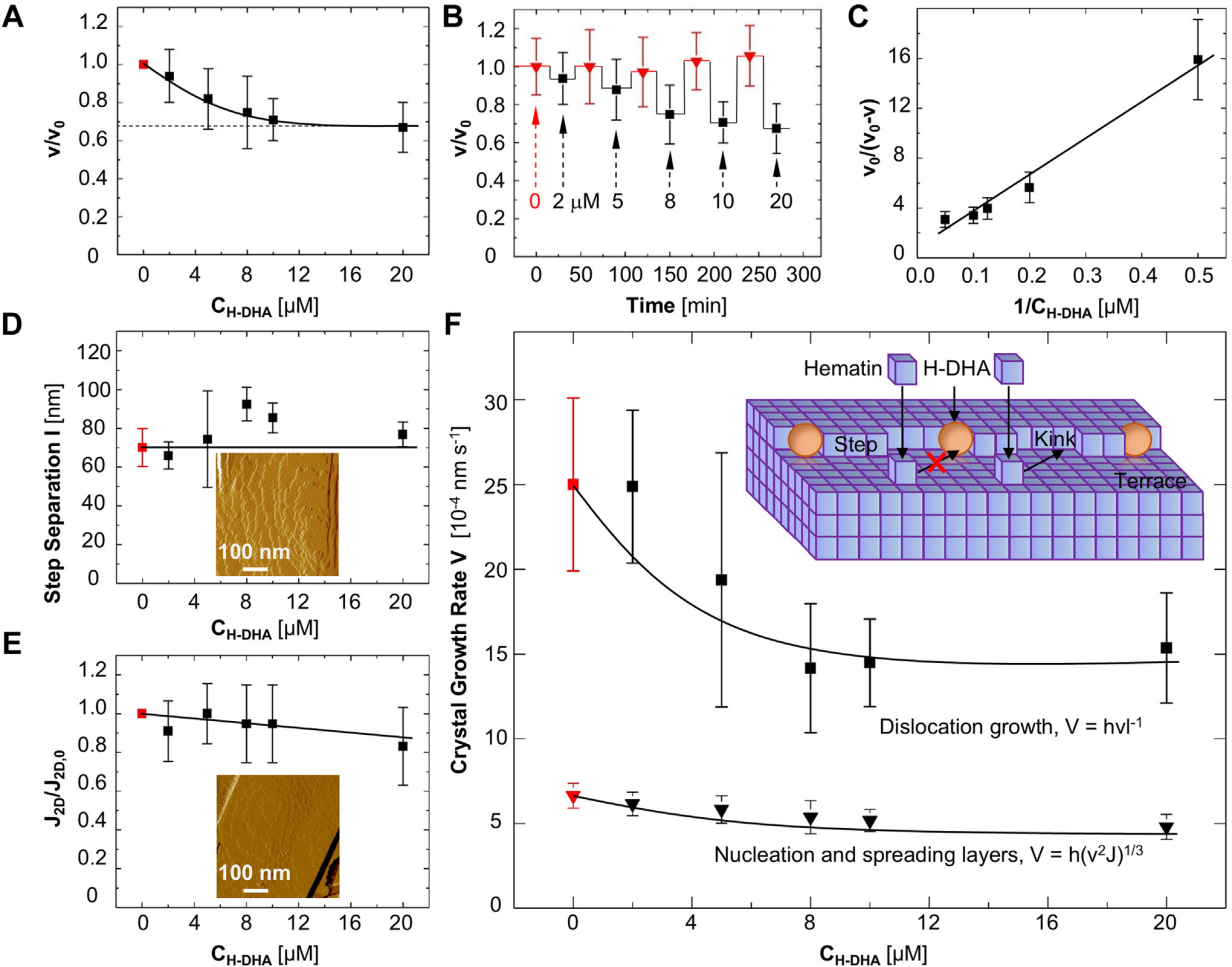
**Time-resolved *in situ* AFM analysis of inhibition of β-hematin growth by H-DHA.***A*, step velocity, *v*, in the [001] direction at increasing H-DHA concentrations, relative to *v* in the absence of the drug, *v*_*o*_. Error bars indicate standard deviations for the slopes of displacement evolutions as shown in Figure 2*D*. *B*, reversibility of H-DHA inhibition of hematin at C_H_ = 0.25 mM. The velocity *v* of steps exposed to solutions with no inhibitor, *red triangles*, initially and between exposures to 2, 4, 8, 10, or 20 μM H-DHA, *black squares*. *C*, linearized step velocity in reciprocal coordinates. R^2^ = 0.85. *D*, separation between steps at several H-DHA concentrations. Error bars indicate standard deviation from the averages of five measurements. *E*, rate of two-dimensional nucleation, *J*_*2D*_, relative to that in the absence of any inhibitor, *J*_*2D,0*_, as a function of H-DHA concentration. Error bars indicate standard deviations from the averages of five measurements. *F*, the growth rates of crystals *V* growing by layers generated by dislocation or 2D nucleation. Error bars indicate standard deviation from the averages of five measurements.

We employ time-resolved *in situ* AFM to monitor how new layers nucleate and grow in the presence of H-DHA. We observe that H-DHA inhibits step growth in the [001] direction of the **β**-hematin (100) face by circa 30% (Figs. 3*A*, S4*A*) at a concentration of H-DHA $C_{H-DHA}$ = 10 μM. Notably, the parent drug, DHA, does not inhibit the step motion (Fig. S4*C*). Importantly, the degree of inhibition does not increase when adjusting $C_{H-DHA}$ as high as 20 μM. Growth suppression that saturates at a certain inhibitor concentration is a signature behavior of kink blockers, modifiers that adsorb to a fraction of the kinks and hinder access of the solute to them (Fig. 3*F*, inset); as v scales with the number of free kinks, blocking of the kinks results in slower v. Full blockage of all kinks is not reached at any concentration of the inhibitor owing to the kink dynamics, whereby kinks are continuously generated on a time scale comparable to that of inhibitor adsorption (40). A closed form expression for this inhibition mode presents the correlation between v and $C_{H-DHA}$ as $v=v_{0}-\left(v_{0}-v_{\infty }\right)\frac{K_{L}C_{H-DHA}}{1+K_{L}C_{H-DHA}}$, where $v_{0}$ and $v_{\infty }$ are the step velocities in a pure solution and at the highest inhibition, respectively, and $K_{L}$ is the Langmuir constant for adsorption at kinks (40). A crucial assumption of the kink blocking model of inhibition is compliance with the assumptions of the Langmuir adsorption model, among which is that inhibitor adsorption is reversible. Tests in which we expose **β**-hematin crystals to alternating solutions without inhibitor and with increasing inhibitor concentrations reveal that adsorption is indeed reversible: we observe that v resumes its initial value once the inhibitor is no longer present (Fig. 3*B*). The $v\left(C_{H-DHA}\;\right)$ correlation linearizes in reciprocal coordinates ${\left(v_{0}-v\right)}^{-1}$ *versus* ${C_{H-DHA}}^{-1}$, ${\left(v_{0}-v\right)}^{-1}={\left(v_{0}-v_{\infty }\right)}^{-1}+{\left[\left(v_{0}-v_{\infty }\right){K_{L}C}_{H-DHA}\right]}^{-1}$. The data for inhibition of hematin step growth by H-DHA are consistent with the kink blocking model (Fig. 3*C*) and support the conclusion that H-DHA inhibits **β**-hematin step growth by blocking the kinks along the steps (Fig. 3*F*, inset). H-DHA shares the kink blocking mode of action with several other antimalarials and their metabolites: mefloquine, amodiaquine, H-ARS, and H-ART (26, 29). These four inhibitors, however, are substantially stronger kink blockers and suppress **β**-hematin step velocities by up to 60% (26, 29).

Mass preservation dictates that the ability of a **β**-hematin crystal to sequester hematin from a solution is determined by the growth rates V of its faces, which, in turn, equals the product of the step velocity v and the step density. If new layers are generated by dislocations and a single dislocation is responsible for the growth, the step density is uniform over a large section of the crystal face and $V=vhl^{-1}$, where l is the average separation between steps and $l^{-1}$ is the step density (58). For growth by 2D nucleation of new layers, with islands that are randomly distributed along the surface and merge as they grow, $V=h\;{\left(v^{2}J_{2D}\right)}^{1/3}$, where $J_{2D}$ is the rate of 2D nucleation of new layers (51, 59). AFM monitoring reveals that both l and $J_{2D}$ are roughly independent of H-DHA concentration (Figs. 3, *D* and *E*, S4).

The correlation of v, l, and $J_{2D}$ with $C_{H-DHA}$ indicate that H-DHA only slightly inhibits the growth of **β**-hematin crystals. If the impact of this metabolite on heme detoxification were limited to this mild suppression of classically growing hematin crystals, then DHA and all drugs of the ART family that convert to DHA in the host body, would be poor suppressors of heme detoxification and inefficient killers of trophozoite-stage parasites. To reconcile this conclusion with the known H-DHA activity on the parasite life stages during which hematin crystallization helps the parasite survive the accumulation of heme owing to hemoglobin digestions (30), we tested if H-DHA may affect hematin crystal nucleation (60) and inhibit nonclassical modes of crystal growth that may activate at higher hematin concentrations.

### Impact of H-DHA on **β**-hematin crystal nucleation?

**Figure 4:**
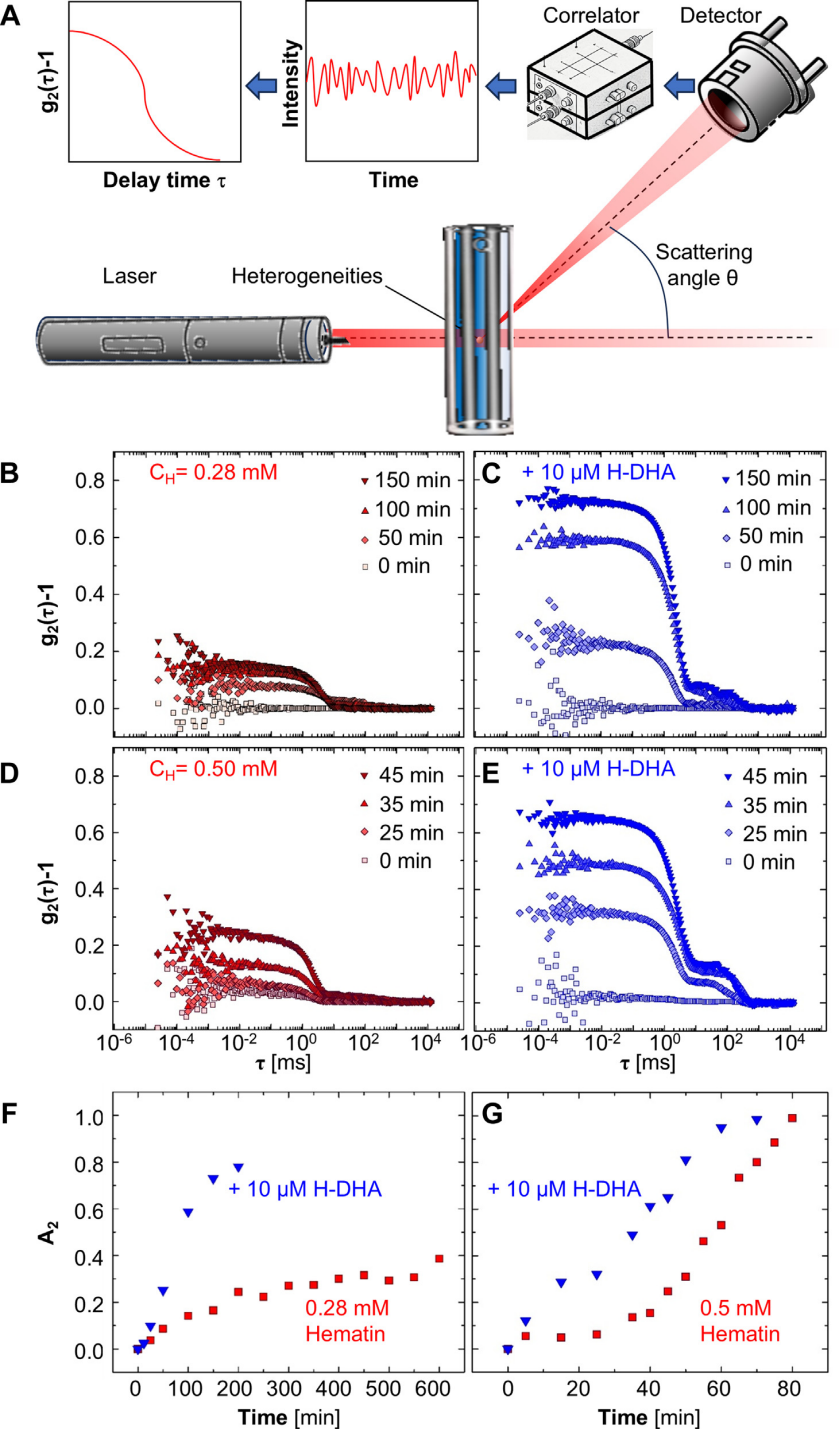
**DHA enhances β-hematin crystal nucleation.***A*, schematic of dynamic light scattering (DLS). *B–E*, representative DLS intensity autocorrelation functions at the following conditions: *B*, C_H_ = 0.28 mM, C_H-DHA_ = 0. *C*, C_H_ = 0.28 mM, C_H-DHA_ = 10 μM H-DHA. *D*, C_H_ = 0.50 mM, C_H-DHA_ = 0. *E*, C_H_ = 0.50 mM, C_H-DHA_ = 10 μM H-DHA. *F* and *G*, evolutions of amplitudes of correlation functions, A_2_ in the absence and presence of H-DHA for two different C_H_. *F*, 0.28 mM. *G*, 0.5 mM.

We monitored hematin nucleation in the presence of H-DHA using dynamic light scattering (DLS). This method detects heterogeneities in the solution, *i.e.*, objects with greater refractive index, from the light that they scatter (Fig. 4*A*). As these objects diffuse within the illuminated volume, the scattered light intensity fluctuates. The DLS method relies on the autocorrelation function $g_{2}\left(\tau \right)$ of the scattered light. The characteristic delay time τ of decay of $g_{2}$ represents the characteristic time of the intensity variations, and thus of diffusion of the scatterers (61, 62). At $C_{H}$ = 0.28 or 0.50 mM the autocorrelation function $g_{2}\left(\tau \right)$ is initially flat, indicating the lack of any heterogeneous objects in the hematin solution (Fig. 4, *B*–*E*). At later times, a shoulder in $g_{2}$ appears and grows indicating increasing numbers and sizes of the scatterers. The shoulders grow much faster in solutions with higher $C_{H}$ (Fig. 4, *B* and *D*), suggesting that the scatterers are domains of hematin condensed phases. The characteristic diffusion times $\tau_{2}$ of $g_{2}$ are circa 1 ms, corresponding to the diffusion of particles several hundred nanometers in size. These $\tau_{2}$ do not extend longer than circa 10 ms (Fig. 4, *B* and *D*), indicating that the sizes of the hematin aggregates illuminated by the incident beam are limited to circa 1 μm. Such behavior is common in DLS measurements of growing objects of density greater than that of the host solution, which sediment above a threshold size. We conclude that the scatterers are **β**-hematin crystals (notably, both tested $C_{H}$ exceed the crystal solubility $C_{e}$ = 0.08 μM), whose density is 1.45 g cm^−3^ (12), and the increase of the $g_{2}\left(\tau \right)$ shoulder characterizes their nucleation and growth to circa 1 μm size.

The addition of H-DHA to the hematin solutions leads to substantially faster increase in the $g_{2}\left(\tau \right)$ shoulders at both $C_{H}$ (Fig. 4, *B*–*E*), indicating that H-DHA strongly enhances the nucleation of **β**-hematin crystals. This conclusion is affirmed by the amplitudes $A_{2}$ of the $g_{2}\left(\tau \right)$ functions; $A_{2}$ roughly equals the drop of the correlation function between short and long delay times τ. These $A_{2}$ values increase with time much faster in the presence of H-DHA than in its absence (Fig. 4, *F* and *G*). Notably, H-DHA boosts **β**-hematin nucleation stronger than H-ART. The highest ratio between $A_{2}$ in the presence of H-DHA to $A_{2}$ in its absence is about four-fold, at 40 min after start of nucleation monitoring (Fig. 4*G*), compared to a two-fold ratio between the equivalent $A_{2}$ values for H-ART (Fig. 2*H* in Ref. (25)).

**Figure 5:**
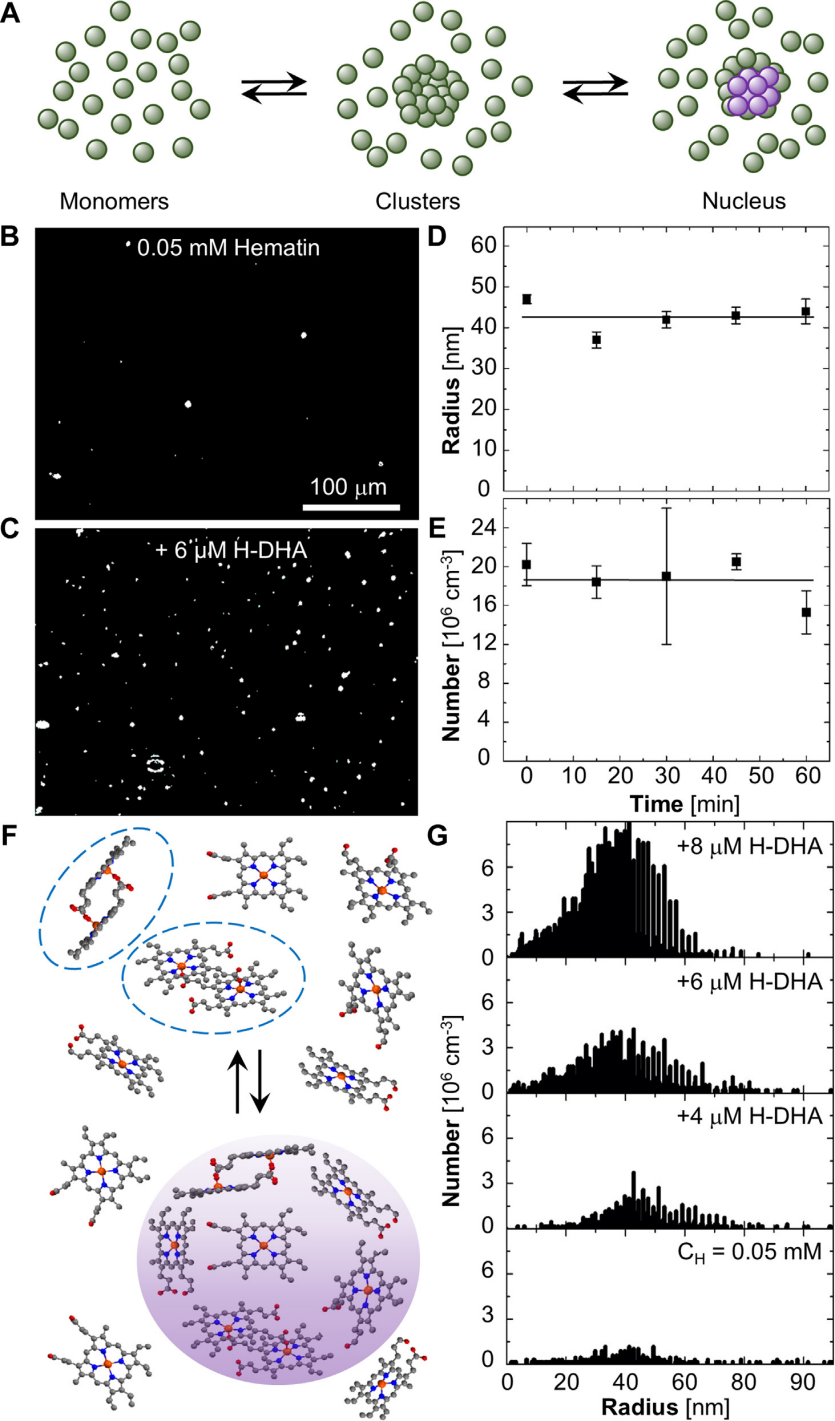
**The mesoscopic hematin-rich clusters.***A*, schematic of crystal nucleation hosted by a disordered precursor. Color coding: green, disordered; purple, crystalline. *B* and *C*, representative Brownian microscopy micrographs. Light speckles correspond to hematin clusters. *B*, at C_H_ = 0.05 mM. *C*, at the same C_H_ after adding 6 μM of H-DHA. *D*, size of the hematin clusters as a function time. *E*, number of hematin clusters as a function of time. In (*D*) and (*E*), error bars indicate standard deviations from the averages of five measurements. *F*, schematic of the formation mechanism of mesoscopic hematin-rich clusters (highlighted with a *purple* shading) owing to the accumulation of hematin dimers (*blue dashed ovals*). Color coding: C atoms shown in *grey*; N, *blue*; Fe, *orange*; O, *red*; H atoms are omitted for clarity. *G*, size distributions of hematin clusters in the presence of 0, 4, 6, and 8 μM of H-DHA, with constant C_H_ = 0.05 mM.

To understand the mechanism employed by H-DHA to boost the nucleation rate of **β**−hematin, we recall that in many cases crystal nucleation from solution proceeds nonclassically, in two or more steps (63, 64). In common two-step pathways, the first step comprises the formation of a precursor, which, in the second step, hosts and facilitates the nucleation of crystals (Fig. 5*A*) (65, 66, 67, 68). To test for the presence of potential nucleation precursors and to explore their response to the presence of H-DHA, we used Brownian microscopy. In this method, a laser illuminates a hematin solution sample (Fig. S5). The light scattered by heterogeneities is collected by a camera (Figs. 5, *B* and *C*; S5). The trajectories of the individual particles are processed to evaluate their diffusion coefficients, which correlate with size *via* the Stokes-Einstein relation (69, 70). In the absence of hematin, no heterogeneities are detected, which classifies the scatterers as hematin aggregates. To exclude **β**-hematin crystals as a potential cluster phase, these tests employed $C_{H}$ = 0.05 mM, which is below the crystal solubility $C_{e}$ = 0.08 mM.

The sizes and concentrations of the observed particles appear steady within the measurement error over times spanning an hour (Fig. 5, *D* and *E*). The lack of particle growth eliminates dense liquids (71, 72) as the constituent phase. Their small sizes, narrow size distributions, and strong light scattering suggest that they are not amorphous agglomerates. On the other hand, the observed behaviors are typical of mesoscopic solute-rich clusters (67, 73, 74, 75, 76, 77) that have been observed with systems ranging from proteins to large organic molecules and inorganic salts. The clusters were found to represent a unique phase, whose domain size is dictated by the dynamics of formation, decay, and diffusion of solute oligomers, *e.g.*, dimers (Fig. 5*F*) (74, 75, 76, 78, 79). The hematin particles comply remarkably well with the predictions of the models developed for mesoscopic solute-rich clusters.

The addition of increasing concentrations of H-DHA to solutions of constant hematin concentration, 0.05 mM, induces a dramatic increase in the cluster concentration (Figs. 5*G*, S6*B*). The average cluster size of circa 40 nm, however, is unaffected by the added H-DHA (Figs. 5*G*, S6*A*). Both behaviors concur with the prediction of the dimer model of cluster formation, according to which the cluster size is unaffected by the thermodynamic parameters of the solution (70, 74, 78, 79). We tentatively attribute the enhanced hematin-rich clustering in the presence of H-DHA to the intermolecular repulsion between H-DHA and hematin molecules. We propose a tentative mechanism for this repulsion. Both hematin (Fig. 5*F*) and H-DHA (Fig. 1*D*) carry two carboxyl groups with pK_a_ ≈ 5. Although the amount of water dissolved in the crystallization solvent, CBSO, is below its solubility, in the majority component, octanol (80), it is feasible that nanoscopic clusters of water molecules may assemble (81), especially around the polar carboxyl groups of hematin and H-DHA. It has been suggested that the water-enriched regions wrapped around the hematin carboxyl groups may crucially enable their coordination to the iron and hydrogen bonding to other carboxyl groups that support the **β**-hematin structure (48, 82). The high dielectric constant of water in these clusters would stabilize the deprotonated state of the carboxyl groups, leading to negative charges on both hematin and H-DHA. With the repulsion between the carboxyl groups of hematin and H-DHA, H-DHA acts as crowder, which restricts access of hematin to substantial volumes of the solution, *via* steric hindrance, thus increasing the solute’s chemical potential and driving dimerization and cluster formation. This activity of H-DHA is similar to how a conventional crowder, polyethylene glycol, enhances mesoscopic clustering of the protein p53 (78, 79). All-atom molecular dynamics simulations to test the details of this proposed mechanism are currently underway (57, 83).

The strong increase in the concentration of mesoscopic hematin-rich clusters by H-DHA (Fig. 5*G*) runs parallel to the effects of H-DHA on **β**-hematin crystal nucleation (Fig. 4, *B*–*G*), suggesting that enhanced cluster formation is the mechanism by which H-DHA boosts **β**-hematin nucleation. A discrepancy appears, however, between this finding, which should correlate with enhancement, and not suppression, of heme detoxification by H-DHA, and the high efficacy of the parent drug, DHA, as a suppressor of trophozoite-stage parasites and its potency as an antimalarial drug (21, 22, 23, 84). To resolve this controversy, we explore whether H-DHA may mobilize additional pathways to inhibit **β**-hematin growth which operate at elevated concentrations of hematin and H-DHA.

### Dual action of H-DHA to irreversibly inhibit **β**-hematin growth

Hemoglobin is supplied to the digestive vacuole of *P. falciparum* discretely, by hemoglobin-loaded transport vesicles that merge with the digestive vacuole (85). Hence, the concentration of hematin, the by-product of hemoglobin digestion, varies between a high value, occurring shortly after the arrival of a vesicle, and a low value preceding the delivery of the next hemoglobin serving. We tested whether the variability in hematin concentration may invoke distinct mechanisms of **β**-hematin inhibition by exposing growing crystals to elevated concentrations of hematin, 0.50 mM, and H-DHA, 10 μM, which are both within the ranges measured in parasites (6, 39).

**Figure 6:**
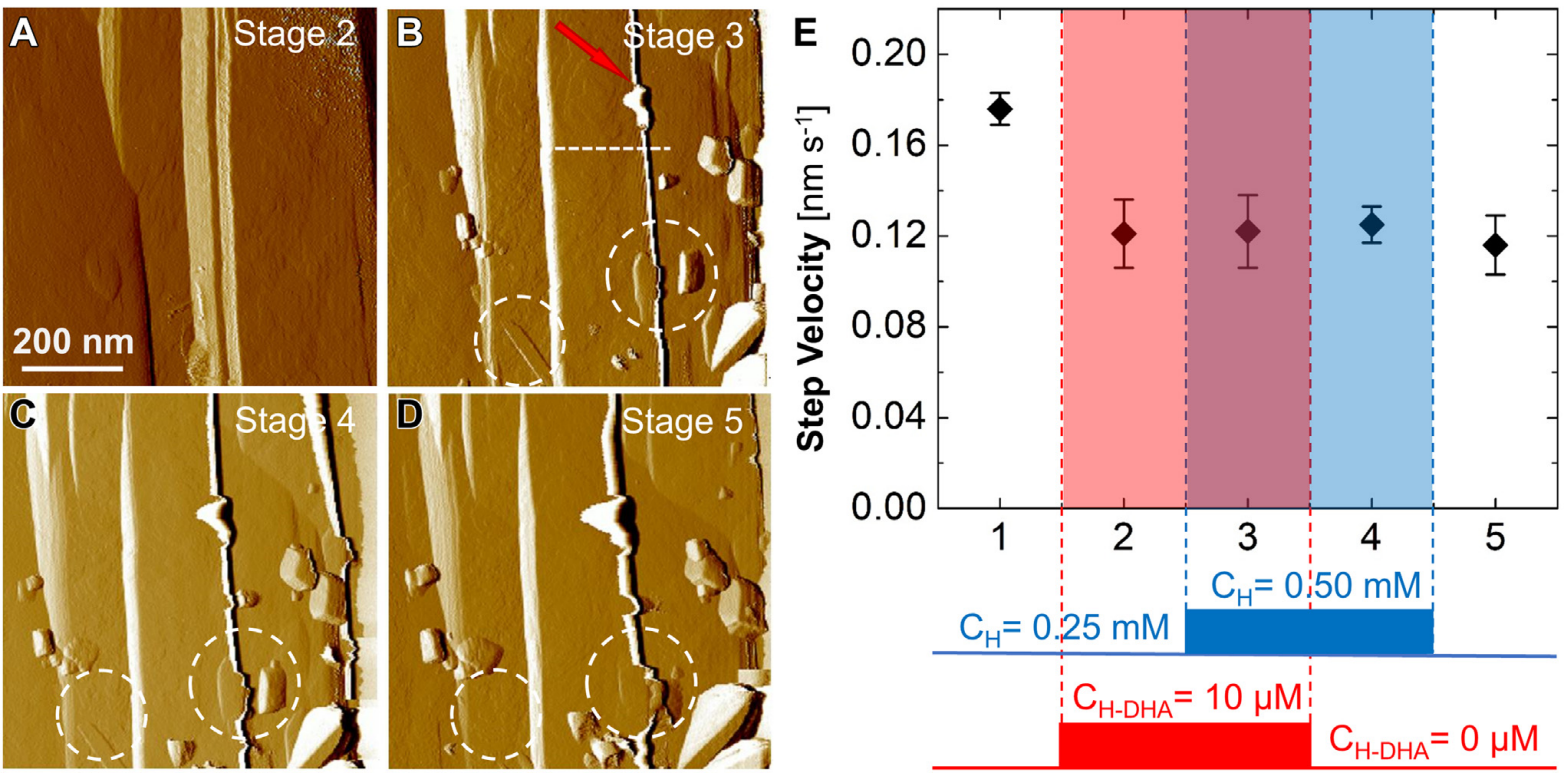
**Irreversible inhibition of β-hematin crystal growth by H-DHA.***A–D*, *in situ* AFM images of a hematin crystal (100) surface growing at hematin concentrations 0.25 and 0.50 mM and in the absence and presence of 10 μM H-DHA. *A*, classical inhibition by H-DHA as in Figure 3. *B*, nanocrystals and a macrostep (indicated with a *red arrow*) at C_H_ = 0.5 mM and C_H-DHA_ = 10μM. Surface profile along *dashed line* shown in Figure S8. *C*, nanocrystals, highlighted in *white circles*, are buried under growing steps. *D*, nanocrystals and macrosteps persist after C_H_ and C_H-DHA_ resume their initial values. *E*, AFM measurements of step velocity at surface segments between the macrosteps at different combinations of C_H_ and C_H-DHA_. Step velocity in the absence of drug (stage 1), in the presence of 10 μM H-DHA (stages 2 and 3), at increased heme concentration, 0.50 mM, in the presence (stage 3), and in the absence (stage 4) of drug; and again, at the initial heme concentration, 0.25 mM, in the absence of drug (stage 5). Error bars indicate standard deviation from the averages of five measurements of step displacement.

The addition of H-DHA to a 0.25 mM hematin solution in contact with **β**-hematin crystals preserves the crystal surface, *i.e.*, AFM observations reveal 2D islands and sequences of parallel steps (Fig. 6*A*). An increase in hematin concentration to 0.50 mM, however, engenders two dramatic behaviors. First, numerous sharp-faceted crystallites with sizes ranging from several tens to 200 nm land on the surface at random locations with random orientations relative to the underlying crystal structure (Fig. 6*B*). The crystallite morphology is akin to that of **β**-hematin, and we tentatively identify this species as a hematin nanocrystal. Their abundance correlates with the enhanced nucleation of **β**-hematin at high concentrations of hematin and H-DHA (Fig. 4, *B*–*G*). The crystallites grow more slowly than the larger underlying crystal and, over time, they are overgrown and submerged under the **β**-hematin steps (Fig. 6, *B*–*D*) that continuously nucleate and spread at these concentrations of H-DHA (Fig. 3, *A*, *D*, *E*). The random orientations of adsorbed crystallites exclude seamless fusion with the lattice of the larger crystal that engulfs them. The mismatch between the two lattices would likely cause substantial elastic strain (86).

The second dramatic behavior is the appearance of macrosteps as high as hundred lattice parameters on the surface (Figs. 6, *B*–*D*, S8). Judging from their separations from landed crystallites, the macrosteps are immobile. Over time they thicken, likely by associating elementary steps that advance towards them on the adjacent surface stripes. The distinguishing criterion between macrosteps and step bunches is whether the constituent elementary steps retain their individuality by staying apart further than the step width (87, 88, 89). While step bunches have been observed during **β**-hematin growth in the presence of pyronaridine, a quinoline class drug (25, 29), this appears to be the first observation of **β**-hematin macrosteps. Numerous analytical and computational models of step dynamics argue that macrosteps form as a result of attractive interactions between steps, which create a positive feedback loop that slows down and brings together steps in close proximity (59, 90, 91). We identify two potential interactions that operate during **β**-hematin growth and may induce macrosteps. First, hematin from the solution reaches the steps after initially adsorbing on the terraces between them (28). The constrained 2D diffusion fields to the steps lead to competition for supply between adjacent steps and slower growth of closely spaced steps (Fig. S9) (92). A second contributing factor is the strain due to the lattice mismatch with the engulfed hematin crystallites that is known to enhance macrostep formation (91).

The two consequences of elevated hematin and H-DHA concentrations—hematin nanocrystals and macrosteps—introduce a potent dual-mode. Suppression of **β**-hematin growth. The lattice strain due to the incorporated nanocrystals increases the chemical potential of hematin in the crystals, lowers the supersaturation, and suppresses step growth (Fig. 6*E*). In contrast to steps inhibited to about the same circa 30% by H-DHA kink blocking (Fig. 3*A*), growth suppression remains even after H-DHA is removed from the solution as long as the strain persists (Fig. 6*E*). The fallout from the macrostep formation is more severe. The macrosteps do not grow. Another way in which they stall growth is that they create permanent barriers between sections of the **β**-hematin surface. If a dislocation outcrops in any of these sections, the steps it generates cannot spread to adjacent sections, which are thus limited to layer generation by 2D nucleation. Growth by 2D nucleation is less efficient and only supports growth slower by circa three-fold than dislocation-driven growth, as shown in Figure 3*F*. The synergistic effects of strain and macrosteps generate an approximate fourfold suppression of **β**-hematin growth. Since macrosteps generated at high hematin and H-DHA concentrations remain in H-DHA-free solutions, even at moderate $C_{H}$ (Fig. 6*D*), this mode of inhibition will also persist even in adduct-free solutions, *e.g.*, after the parent drug is cleared from a patient’s organism and from the parasite (93). This irreversible mode of inhibition is unique for H-DHA. Previous studies with chloroquine and mefloquine demonstrate that after these drugs are removed from the system, crystal growth resumes with its rate prior to drug introductions (25).

We envision that over longer times, under conditions of moderate hematin concentration and no H-DHA, crystal growth may return to its normal unsuppressed rate. If no crystallites nucleate, the strain due to the previously incorporated crystallites would dissipate as the crystals accumulate newly grown layers *via* classical mechanisms (44, 94). Macrosteps may also fall apart even though the exact mechanism is less understood (88, 89). The crystal growth rate V driven by 2D nucleation is of order 10^−4^ nm s^−1^. Thus, the accumulation of several crystal layers would take at least several hours. This time period extends several-fold the time of action of artemisinin-class drugs, whose concentration in the human body drops by half within 1 to 3 h after administration and which are fully eliminated within 10 h (95, 96).

While alternative ART-class adducts from prior studies (H-ART and H-ARS) were shown to boost nanocrystal nucleation at elevated hematin concentrations (25), they do not evoke macrosteps as we observe here for H-DHA. The unavailability of the potent macrostep pathway to H-ART may be the mechanistic explanation of the weaker suppression of its parent drug, ART, of trophozoite-stage parasites (30). Importantly, both nanocrystals nucleation (97) and macrostep formation (91) are cooperative phenomena whose rates and extents are strong functions of their shared driving parameters, H-DHA and hematin concentrations.

## Conclusion

We find that the adduct of hematin and dihydroartemisinin, the common metabolite of artemisinin-class antimalarials, engages two distinct modes of interaction with hematin crystallization, which is the main constituent of the heme detoxification pathway of malaria parasites. The two modes of action are selected depending on the concentrations of hematin and the adduct. At moderate concentrations, which mimic the conditions in parasite strains resistant to artemisinin-class drugs, the adduct weakly blocks the growth sites on crystal surfaces, does not inhibit two-dimensional nucleation of new crystal layers, and moderately reduces the rate of hematin crystal growth. Furthermore, the adduct promotes crystal nucleation (by boosting the formation of nucleation precursors), an adverse activity that would enhance the rate of heme detoxification in parasites.

At elevated concentrations of hematin, as in parasites sensitive to artemisinin drugs, the adduct’s acceleration of crystallite nucleation leads to their incorporation into **β**-hematin crystals in a manner that suppresses growth, likely by straining the underlying crystal lattice. A second consequence of combining elevated hematin concentrations and the adduct is the generation of numerous macrosteps. The macrosteps do not grow and slow down overall crystal growth by circa fourfold. Growth suppression by incorporated crystallites and macrosteps is irreversible and persists after the adduct is removed from the solution and may extend well beyond the time of action of artemisinin-class drugs, whose half-life in the human body is limited to several hours. Macrostep formation is an inhibition mechanism unique to the hematin-dihydroartemisinin adduct and may be the cause of the substantially greater potency of dihydroartemisinin in suppressing trophozoite-stage parasites. The rates of both crystallite nucleation and macrostep formation strongly depend on adduct and hematin concentrations. These strong dependences tentatively suggest that a moderate two-fold decrease of the hematin and adduct concentrations in resistant parasites (6, 39) allow their survival by excluding these powerful mechanisms of irreversible hematin crystallization inhibition.

A question that our findings highlight is how the hydrogenation of a carbonyl group of artemisinin that converts it to a hydroxyl group (Fig. 1, *B* and *C*) changes the activity of the drug so significantly: it weakens it kink blocking ability and boosts a nonclassical inhibition pathway by strongly enhancing crystal nucleation. This question is a crucial part of the rational design of hematin crystallization inhibitors that have the potential to serve as novel antimalarial drugs. The results presented here provide another element in the data set that may serve as a guide in a strategy of rational inhibitor design.

## Methods

### Materials

The compounds listed below were acquired from Sigma Aldrich: citric acid (anhydrous, anhydrous, ≥99.5%), sodium hydroxide (anhydrous, ≥98%), n-octanol (anhydrous, ≥99%), n-butanol (anhydrous, ≥99%), sodium dithionite, and dihydroartemisinin. Deionized (DI) water was obtained using a Millipore reverse osmosis-ion exchange system (Rios-8 Proguard 2 – MilliQ Q-guard). Porcine hematin was purchased from Chem-Impex, with a purity of 97% as confirmed by HPLC.

### Synthesis of H-DHA

To synthesize H-DHA, hematin powder was dissolved in n-butanol at 45 °C over a period of 1 day and then combined with an aqueous sodium dithionite solution under a flow of nitrogen gas, maintaining the molar ratio of hematin to sodium dithionite at 1:5. The vial was vigorously shaken to ensure thorough mixing of the organic and aqueous phases. Physical mixing of heme (III) in n-butanol with an aqueous solution of sodium dithionite results in a biphasic solution (Fig. S1(i)), where the reducing agent (dithionite) converts heme (III) to heme (II) through a redox reaction (Fig. S1, inset. This process reduced the hematin to heme (II), causing the organic phase to rapidly turn a raspberry color. A mixture of the drug dissolved in n-butanol is then injected into this solution, followed by shaking (Fig. S3(ii)). Within 30 s, a color change in the organic phase indicates that DHA is activated, leading to the formation of H-DHA *via* the reaction (Fig. S1(iii)). This reaction took place at ambient temperature and was completed in 15 min, with a molar ratio of hematin to artemisinin set at 1:2. The synthesis method for H-DHA mirrors the one used for H-ART and H-ARS (25, 26), with the only difference being the substitution of DHA for ART.

### Purification of H-DHA

The organic layer was filtered using a 0.2-μm membrane prior to injecting it into an HPLC instrument. This setup included a C18 preparative chromatography column alongside dual UV-Vis detectors, with their detection wavelengths set at 215 and 370 nm for identifying the unreacted parent drug (DHA), and heme-adduct compounds (H-DHA), respectively. The mobile phase was a mixture of 0.1% formic acid in deionized water, acetonitrile, and methanol with a gradient method. The flow rate was set at 1 ml min^−1^, and the injected volume was 0.5 ml. Finally, we dried the sample under high vacuum to produce a powder.

We saw that H-DHA has absorbance peaks at 210 nm (DHA) and 410 nm (hematin). H-DHA absorbance pattern is similar to H-ART (Fig. S2*A*). The HPLC chromatogram displays a prominent peak at a retention time of 10.5 min corresponding to H-DHA (Fig. S2*B*). The parent drugs are effectively separated from the heme-drug adducts, as they appear at distinct retention times of 8.5 and 11.5 min during the elution process. H-ART and H-DHA have similar hydrophobic interactions with the C18 column.

We used X-ray diffraction (XRD) to analyze the powder of the adduct (Fig. S2*C*). The results indicate that H-DHA is not amorphous; rather, the dried product appears in crystalline form. Notably, the XRD pattern for H-DHA differs from that of hematin.

### Mass spectrometry

We used mass spectrometry to determine the molecular weight of the adduct. The mass-to-charge (m/z) ratios of heme and its drug adduct were measured by a Bruker MS Single Q LC-MS. The H-DHA was transferred into the electron spray ionization (ESI) source utilizing electrospray ionization with an offset voltage set at 4000 V. The mobile phase consisted of a 50/50 mixture of water and acetonitrile. For dissolution before ionization, H-DHA was dissolved in either n-butanol or a mixture of methanol, acetonitrile, and water.

### Preparation of **β**−hematin crystals for AFM observations

We added 1 ml of 0.1 M NaOH to 10 ml of octanol in a glass vial of 20 ml, then left the cap open on a heat plate at 60 °C for 1 day. This step was done to increase the pH of the original solvent. This allowed more hematin to dissolve. Afterward, we added 12 mg of hematin and placed the solution in an incubator at 60 °C for 1 day. The vial was sealed to avoid oxidation. After that, we filtered the solution with nylon filters with a pore size of 0.22 μm and its concentration was measured using UV-Vis spectrometry. We used extinction coefficients $\varepsilon_{hematin}=3.1\;\pm 0.1\;{\text{cm}}^{-1}{\;\text{mM}}^{-1}\;\text{at}\;\lambda =594\;\text{nm}$ (27). Subsequently, the solution was diluted to a desired concentration using freshly prepared octanol saturated withcitric bufferat pH to 4.8, close to the pH value in the digestive vacuole of the parasite. Moreover, 4.8 is near the pKa of the two propionic groups of hematin, which is beneficial for crystallization (47). To prepare the citric buffer, we prepared 50 mM citric acid solution and gradually added 0.10 M NaOH to the stirring solution until it reached pH 4.8 (28, 98). Then, we added 5 ml of citric buffer to n-octanol at room temperature and allowed the system to equilibrate for 30 min. The upper layer of the two-phase system was decanted and labeled as CBSO. Next, we placed a scratched glass slide (15 mm diameter) inside a vial, contacting the supersaturated solution. The vial was then stored at room temperature in a dark undisturbed place. After a week, we observed small, needle-like structures, and after 14 days of incubation, we observed hematin crystals.

### *In situ* monitoring of **β**-hematin growth by AFM

Hematin crystals were grown on glass disks as described above. The density of attached hematin crystals was monitored under an optical microscope. We ensured similar crystal density for all samples to minimize potential depletion of solute and inhibitors due to high crystal number. The glass disks were mounted on AFM sample disks (Ted Pella Inc), and the samples were placed on the AFM scanner.

The temperature of the growth solution within the AFM fluid cell during *in situ* crystal growth was monitored using a copper-constantan thermocouple connected to a temperature controller (SE5010, Marlow Industries Inc). Calibration of the thermocouple was performed using a crushed ice/DI water bath, which was allowed to equilibrate for 30 min. After equilibration, the freezing point was measured at multiple points in the bath with a mercury thermometer (±0.1 °C) to ensure accuracy. The thermocouple tip was positioned in direct contact with the thermometer, and the controller set point was adjusted to ensure a reading of 0.0 °C ± 0.1 deg. The thermocouple was embedded in a brass disk positioned directly beneath the AFM sample. the temperature in the fluid cell stabilized at 26.2 °C ± 0.1 deg, which was above the ambient room temperature of approximately 22 °C, due to the heat generated by the AFM scanner and laser.

For all atomic force microscopy (AFM) experiments, we employed a multimode AFM system (Nanoscope VIII) from Bruker. The AFM images were obtained in tapping mode using BL-AC40TS probes from Oxford Instrument (Silicon nitride probe, coated with Cr/Au, having a spring constant of 0.09 Nm^−1^) at a tapping frequency of 32 kHz. The image sizes ranged from 500 nm to 5 μm with scanning rates between 1 and 2.1 s^−1^. We used height, amplitude, and phase imaging modes. The images we captured consisted of 256 scan lines and were oriented based on the crystal being observed. **β**-hematin crystals were cultivated on glass slides. These slides were then affixed to AFM sample disks from Ted Pella Inc., and subsequently positioned within the AFM scanner. Solutions for hematin growth in CBSO, with concentrations ranging from 0.08 to 0.50 mM, were freshly prepared no more than 2 hours before use and were thoroughly mixed just prior to injection. Once the solution was loaded, the system was left undisturbed for 10 to 20 min to allow for thermal stabilization.

Hematin crystals deposited on the sample disks in the AFM cells typically position their (100) faces, which have the largest areas, upwards and expose them to AFM monitoring. To determine the crystallographic directions on a (100) face, we use that the [001] direction is parallel to the long edge of a hematin crystal. The (100) faces are typically less than 800 nm wide, which makes this edge readily detectable by widening the AFM scan size.

### Dynamic light scattering

We used a dynamic light scattering (DLS) instrument to investigate the nucleation of hematin in the presence of H-DHA. The instrument had a laser source with a wavelength of 600 nm. The laser beam was reflected off by several mirrors before reaching the sample, where it was scattered by hematin nuclei within the solution. DLS is based on the principle that smaller particles move more quickly than larger ones, leading to faster fluctuations in the intensity of scattered light. The scattered light from multiple particles creates a speckle pattern that changes over time. A photon counting module measures the time-dependent fluctuations in scattered light intensity. An autocorrelator analyzes the intensity fluctuations to determine the particles diffusion coefficient. The diffusion coefficient is used to calculate the hydrodynamic radius of the particles using the Stokes-Einstein equation.

We used two different concentrations as controls: 0.28 and 0.50 mM. Freshly prepared hematin solutions were filtered through 0.20 μm membranes to remove any large particles. After measuring their concentration with a UV-Vis spectrometer, we diluted them to 0.28 mM and 0.50 mM. We poured 2 ml of the solutions into a cylindrical glass cuvette and placed the cuvette inside the sample holder containing toluene. Toluene was used because its refractive index is similar to that of glass. Then, we opened the shutter to allow the laser beam to hit the cuvette. The laser beam scattered in all directions; however, we adjusted the angle between the cuvette and the detector to 90 degrees to collect light exclusively at this angle. The measurements were conducted at a temperature of 25 °C.

The characteristic diffusion time $\tau_{2}$ for hematin crystallites and the respective amplitude $A_{2}$ (which is proportional to the intensity scattered by the crystallites) were calculated by fitting $g_{2}\left(q,\tau \right)$ with an exponential relation (99, 100) g2(q,τ)−1=[(A2exp(−ττ2))2+ε(τ)]2, where ε(τ) is background noise in the correlation function.

In control experiments, we recorded the autocorrelation functions of light scattered from blank CBSO and CBSO with added 10 μM H-DHA (Fig. S10, *A* and *B*). The autocorrelation functions exhibited no increasing shoulder that would indicate the nucleation and growth of a new phase. At random times the autocorrelation functions in both solutions revealed shoulders that disappeared in the next collected autocorrelation function. We attribute these shoulders to water nanodroplets that form in CBSO and were discussed earlier (98). This hypothesis is supported by data from solution of H-DHA in dry octanol, for which the autocorrelation function revealed no such response (Fig. S10*C*). The amplitudes of the autocorrelation functions recorded from the same solutions with higher frequency (Fig. S10*D*) support the observations that heterogeneities in these solutions are rare. Lastly, the photons scattered from solution of H-DHA in CBSO are substantially fewer than those scattered from a solution containing hematin (Fig. S10*E*). In hematin solutions, **β**-hematin crystals, which have a high refractive index, scatter substantially more light. These measurements reveal that in the absence of **β**-hematin crystals, the signals in both the autocorrelation function and its amplitude are low.

### Characterization of hematin nucleation precursors

To detect and identify potential nucleation precursors, we employed Brownian microscopy (69, 70, 73), implemented in a Particle Metrix ZetaView device, to characterize heterogeneities in hematin solution and their response to H-DHA. A cell channel with an inlet and an outlet is illuminated by a laser with a wavelength of 520 nm (Fig. S5). Solution heterogeneities scatter the incident light. The scattered light is captured by a microscope objective and a digital camera, enabling real-time visualization of the particles. Specialized software monitors and analyzes the movement of individual particles undergoing Brownian motion. Using the Stokes-Einstein equation, the software calculates the hydrodynamic diameter of each particle based on its movement.

To prepare the control, we used a hematin solution as described earlier. To eliminate large particles, freshly prepared hematin solutions were passed through 0.20 μm membranes. We measured the concentration with a UV-Vis spectrometer and diluted it to 0.05 mM. We ensured that the concentration of the hematin control solution stayed below the supersaturation level (0.08 mM) to exclude crystalline particles. Then, we added H-DHA to concentrations of 2, 3, 4, 6, and 8 μM of H-DHA. We loaded the solutions into the cell channel using a 1 ml syringe. After loading, we waited several minutes and monitored the clusters with the instrument’s camera to confirm the absence of flow inside the cell channel. We set the device temperature to 25 °C and adjusted the camera to focus on a cross-section in the middle of the fluid cell. For each measurement, we allowed at least 1 hour to ensure that the size and number of hematin clusters remained consistent. We collected results every 15 min and performed five measurements at each interval. We averaged the results to determine the size and number of clusters. We calculated the error bars using error propagation. Since the instrument reports clusters using the viscosity of water, we re-evaluated the sizes using the viscosity of octanol.

## Data availability

The datasets generated during the current study are available as Supporting Information.

## Supporting information

This article contains supporting information.

## Conflict of interest

The authors declare that they have no conflicts of interest with the contents of this article.
