# Supplementary material for: The hematin-dihydroartemisinin adduct mobilizes a potent mechanism to suppress β-hematin crystallization
Source: J Biol Chem. 2025 May 29;301(7):110310. doi: 10.1016/j.jbc.2025.110310 (PMC12271862; doi:10.1016/j.jbc.2025.110310)
Supplement: Supplementary Information [file mmc1.docx]

Supporting Information for

The Hematin-dihydroartemisinin Adduct Mobilizes a Unique Potent Mechanism to Suppress β-hematin Crystallization

Hamidreza Azargoshasb^1,2^, Huan-Jui Lee^1,2^, David J. Sullivan^3^, Jeffrey D. Rimer^1,2,4^,
Peter G. Vekilov^1,2,4,*^


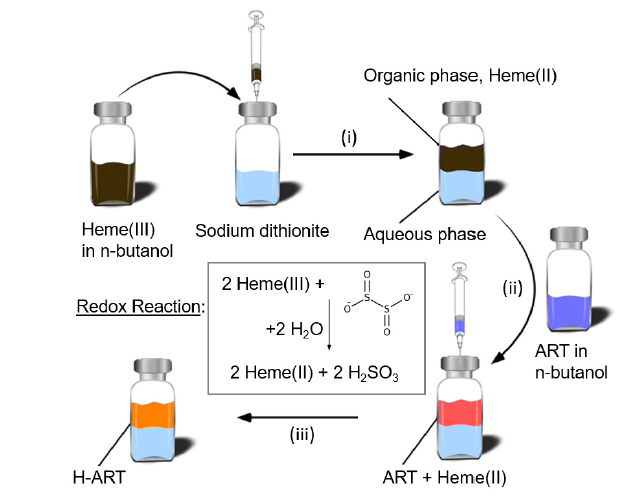


H-DHA

DHA

DHA

25^o^C

**Figure S1.** Steps involved in the synthesis of H-DHA adduct, illustrating the protocol for preparing H-DHA. (i) Physical mixing of heme(III) in n-butanol with an aqueous solution of sodium dithionite results in a biphasic solution, where the reducing agent (dithionite) converts heme(III) to heme(II) through the redox reaction shown in the boxed region. (ii) A mixture of the drug dissolved in n-butanol is then injected into this solution at ambient temperature, followed by shaking. (iii) Within 30 seconds, a color change in the organic phase indicates that DHA is activated, leading to the formation of H-DHA via the reaction.


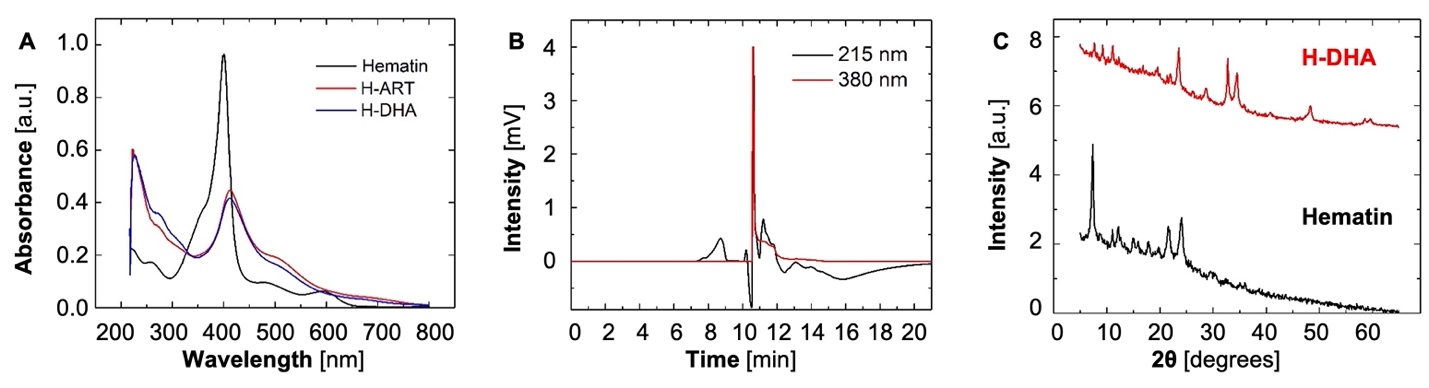


**Figure S2.** Characterization of H-DHA **A.** UV-Vis absorption spectra of hematin (black line), H-DHA (red line), and H-DHA (blue line). The wavelengths of the Soret peaks are shifted in the H-ART and H-DHA spectra from that for hematin. **B.** Chromatograms from high-performance liquid chromatography (HPLC) separations show a major peak for H-DHA at a retention time of 10.5 minutes. Residual parent drug peaks elute at retention times of 8.5 and 11.5 minutes, clearly separated from the heme-drug adducts. **C.** X-ray diffraction (XRD) patterns of heme(III) (black line) and H-DHA (red line).


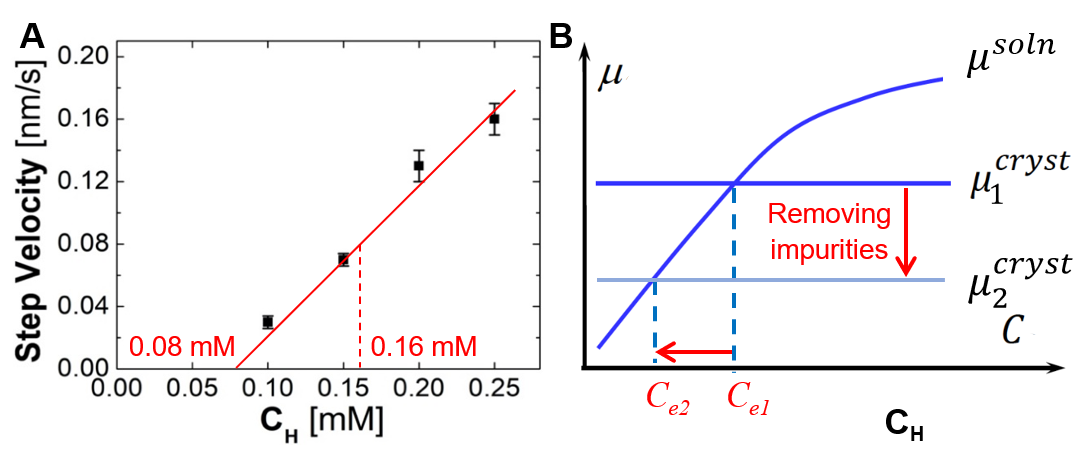


## **Figure S3.** The solubility of β-hematin crystals. **A.** Step velocity as a function of hematin concentration $C_{H}$. The intersection of the linear fit and the $C_{H}$ axis represents the solubility $C_{e}$. Error bars indicate the standard deviations from the mean slopes of the linear correlations as in Fig. 2D. **B.** Schematic illustration of how using higher purity hematin decreases the hematin solubility with respect to crystals $C_{e}$. $\mu^{soln}$ is the chemical potential of hematin in the solution, $\mu^{soln}=\mu_{0}+RT\ln(\gamma_{H}C_{H})$; $\gamma$, activity coefficient. $\mu^{cryst}$ is the chemical potential of hematin in crystals, which is independent of the solution concentration of hematin $C_{H}$. Growth from purer solutions reduces the incorporation of impurities in the crystal and the crystal lattice strain, which lowers $\mu^{cryst}$ from $\mu_{1}^{cryst}$ to $\mu_{2}^{cryst}$. Since $C_{e}$ is $C_{H}$ at which $\mu^{soln}=\mu^{cryst}$, $C_{e}$ decreases from $C_{e1}$ to $C_{e2}$.


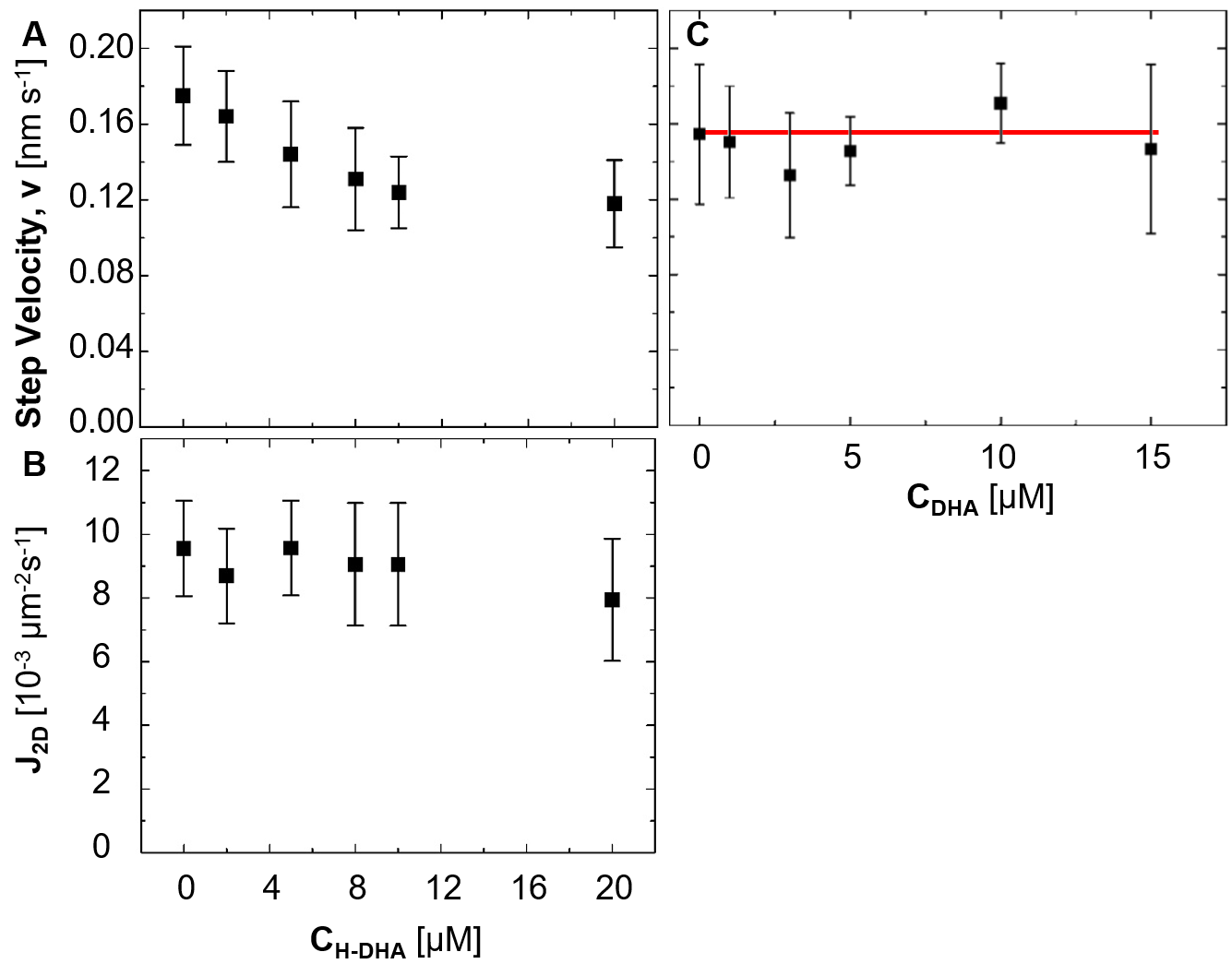


**Figure S4.** **A.** Step velocity *v* in the [001] direction as a function of H-DHA concentration. Error bars indicate the standard deviations from the mean slopes of the linear correlations of step displacement vs time as in Fig. 2D. **B.** Rate of two-dimensional nucleation *J_2D_* as a function of H-DHA concentration. Error bars indicate standard deviation from the averages of five measurements. **C.** Step velocity v in the [001] direction as a function of the concentration of the parent drug DHA. Error bars indicate the standard deviations from the mean slopes of the linear correlations of step displacement vs time as in Fig. 2D. Horizontal line marks the step velocity *v* in solutions with C_H-DHA_ = 0


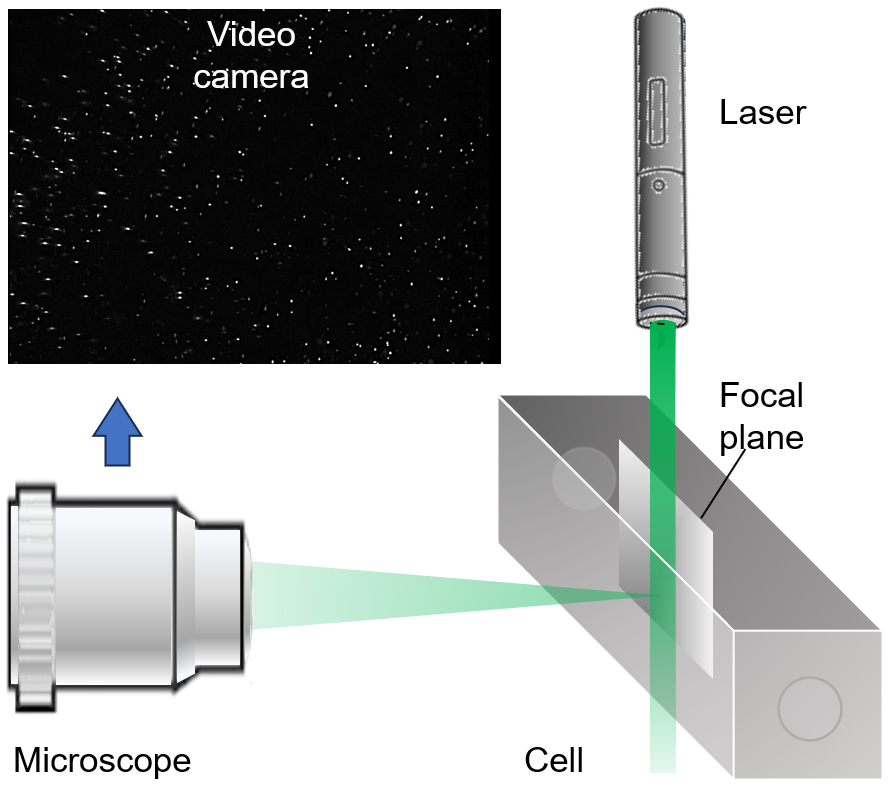


**Figure S5.** Schematic of Brownian microscopy setup.


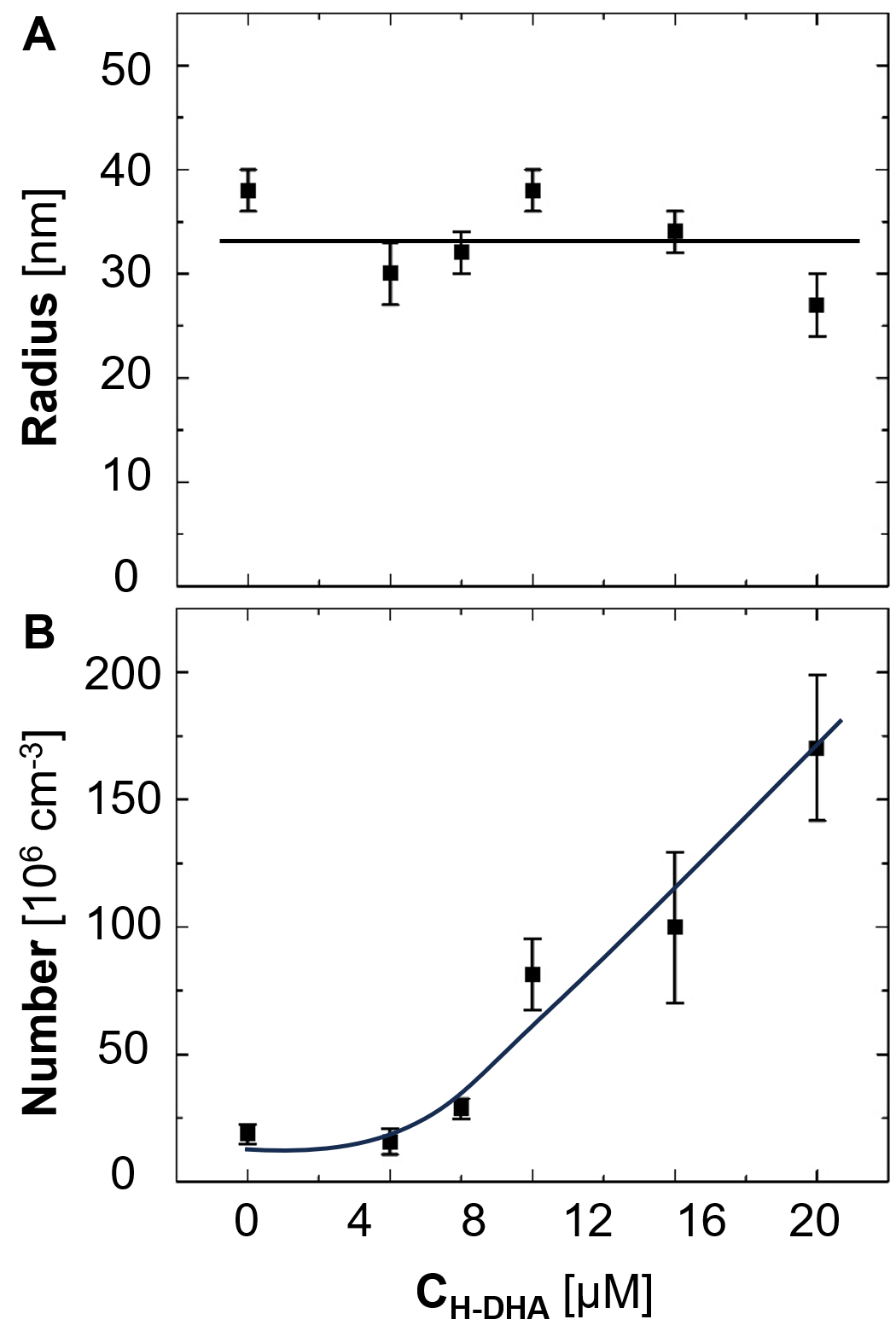


**Figure S6.** Response of hematin clusters to DHA concentration C_H-DHA_. **A.** Size of the hematin clusters as a function of C_H-DHA_. Horizontal line represents the average size. **B.** Number of the hematin clusters as a function of C_H-DHA_. Line is a guide to the eye. Error bars in A and B indicate standard deviation from the averages of five measurements.


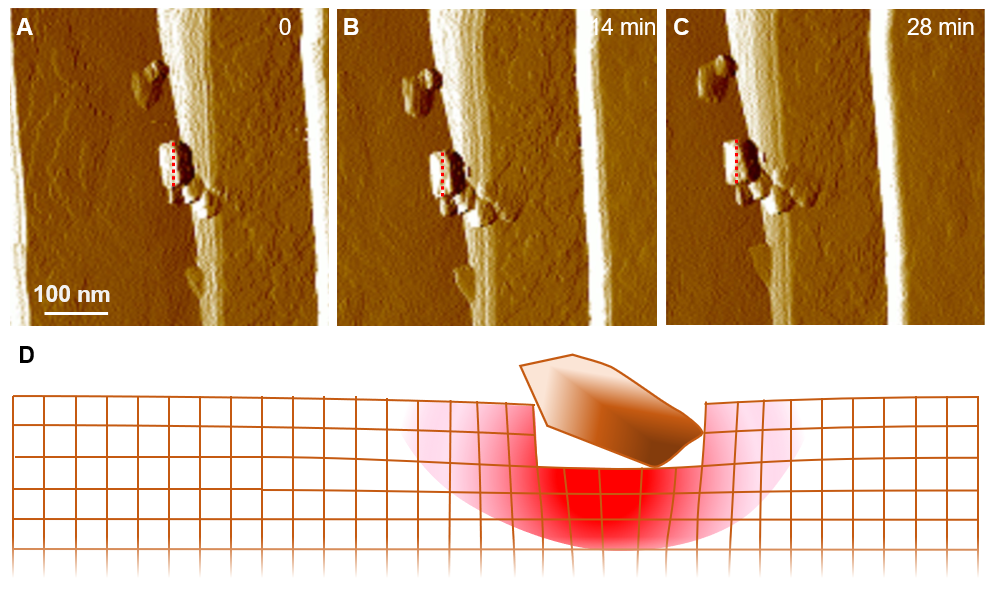


**Figure S7.** Nanoscrystals on the surfaceof a β-hemrtin crysrtal. **A – C.** Expanded view of the nanocrystals in Fig. 6B. The evolution of a nanocrystal growth in the presence of 10 mM H-DHA in stage 3. The length of an individual nanocrystal (indicated with a red dashed line) remains steady at ca. 70 nm over 28 min. **D.** Schematic of lattice strain introduced by the incorporation of a nanocrystal in the lattice of a larger crystal.


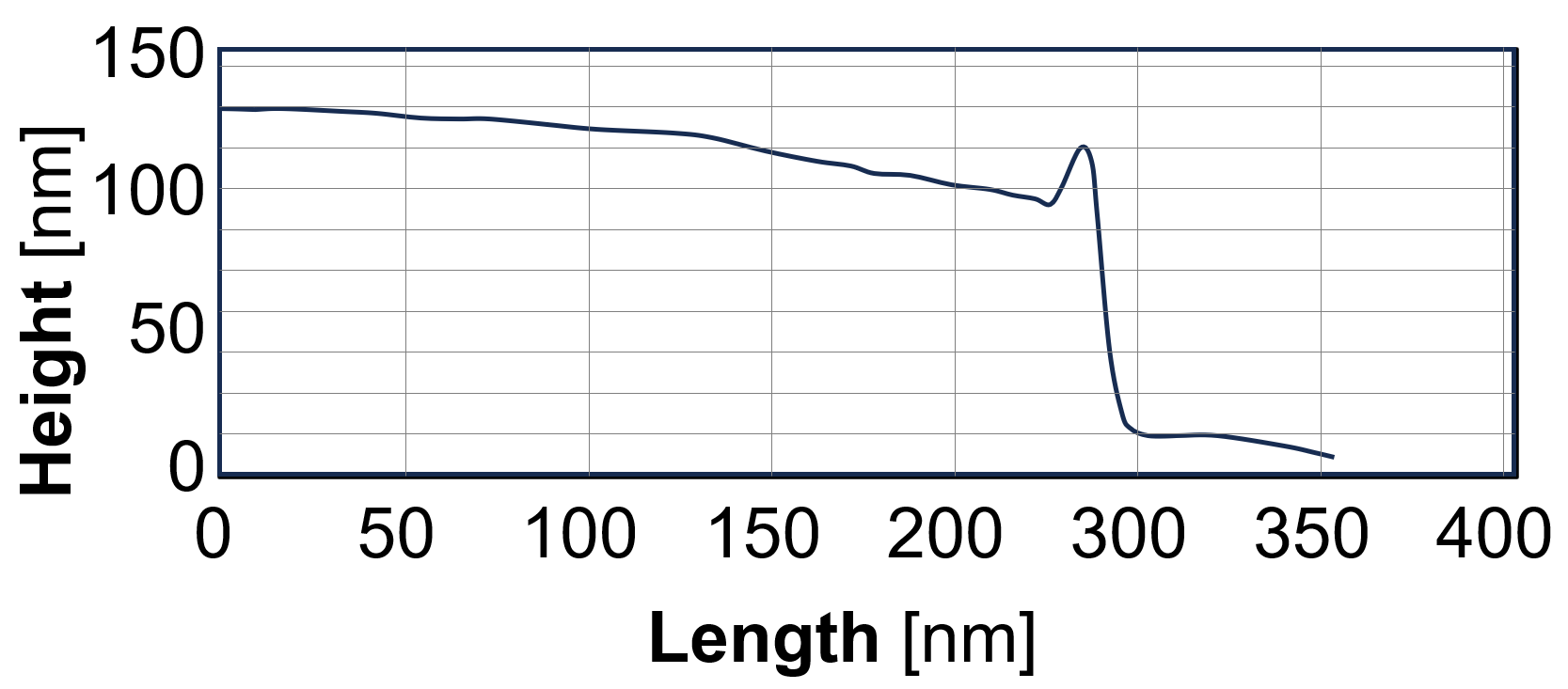


**Figure S8.** Cross section of the crystal surface perpendicular to a macrostep along white dashed contour in Fig. 6B.


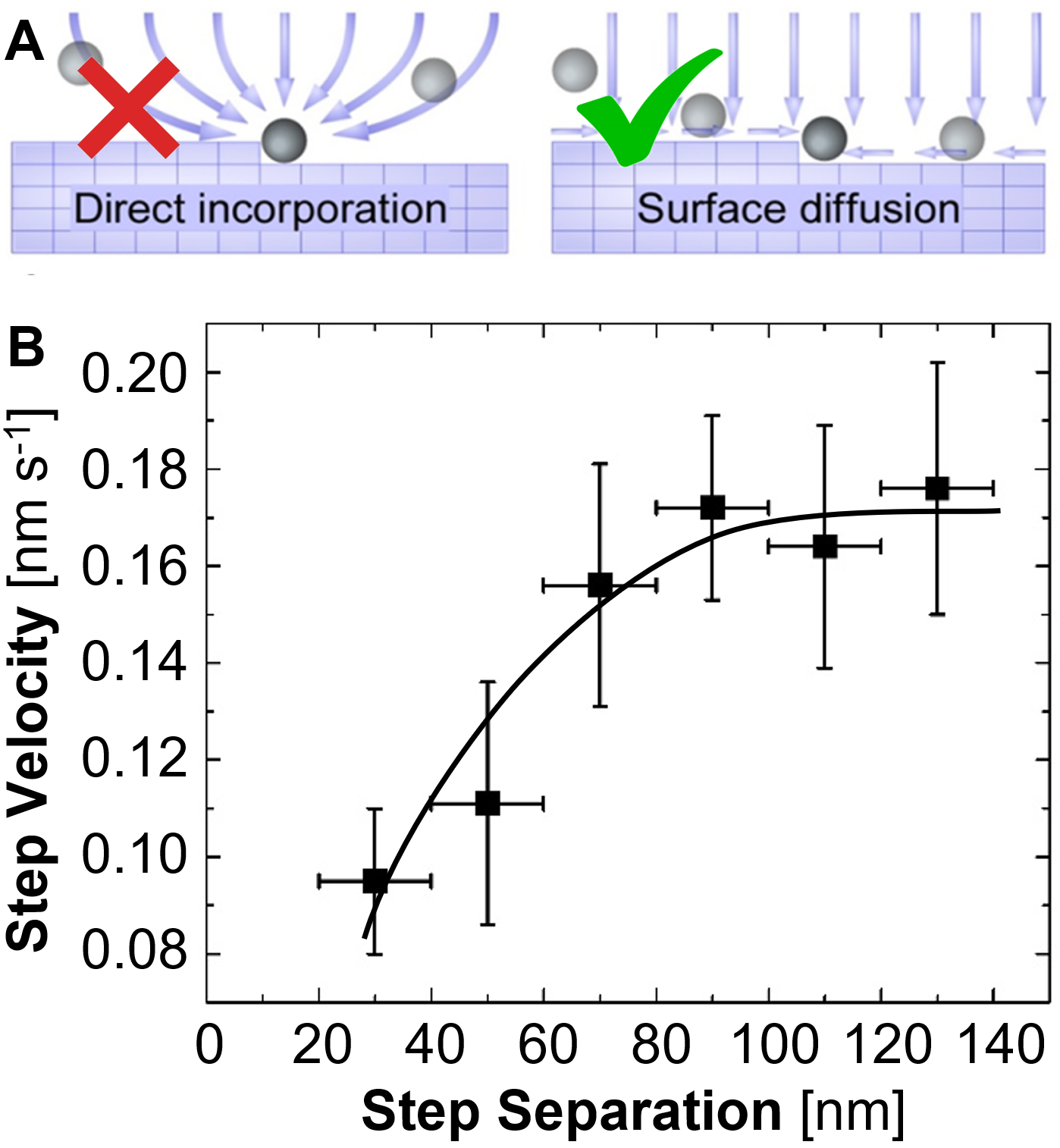


**Figure S9.** The pathway of the hematin molecules from the solution to the steps. **A.** Schematic representations of two pathways for solute supply to the steps: directly from the solution, and after adsorption on the crystal surface followed by diffusion towards the steps. **B.** The correlation between step separation and step velocity. Vertical error bars indicate standard deviation from the averages of five measurements. Horizontal error bars indicate the range of interstep distances. Closely spaced steps grow substantially slower than well-separated steps, indicating that the surface diffusion pathway applies. Solid line is a guide to the eye.


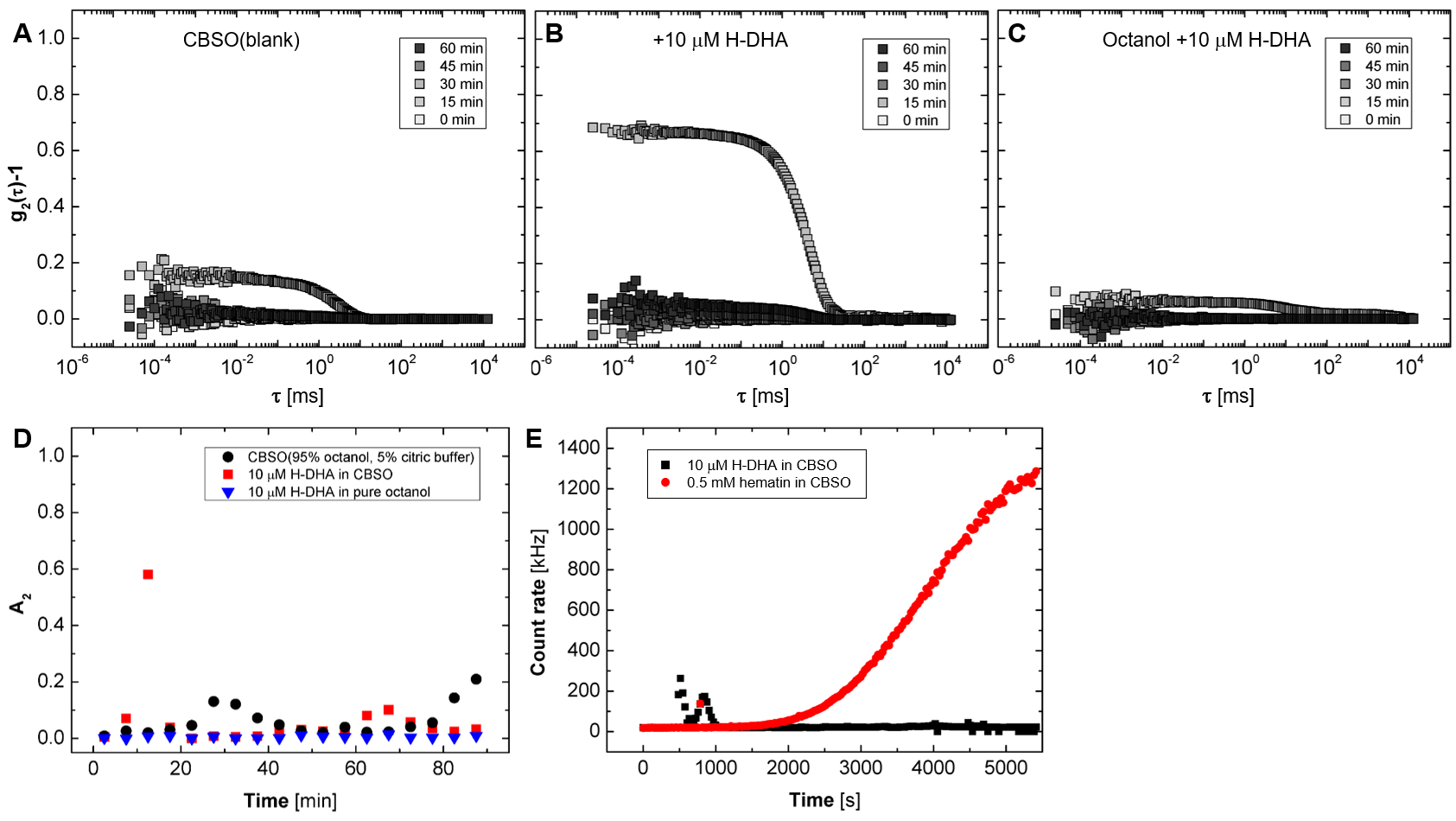


**Fig. S10.** Controls for characterization of β-hematin crystal nucleation by dynamic light scattering. **A – C.** The autocorrelation functions in at time indicated in the plots. **A.** CBSO with no addition of hematin or H-DHA. **B.** CBSO with added 10μM H-DHA. **C.** Dry octanol with added 10 μM H-DHA. **D.** Evolutions of the autocorrelation function amplitudes in the three solutions whose select autocorrelation functions are displayed in A – C. **E.** Numbers of photons scattered during DLS measurements at conditions indicated in the plot. The nucleation of β-hematin crystals in the hematin-containing solution leads to a substantial number of scattered photons.
